# Association between apathy in patients with maintenance dialysis and hospitalization or mortality: a prospective cohort study

**DOI:** 10.3389/fpsyt.2023.1007977

**Published:** 2023-04-20

**Authors:** Yu Fang, Lei Chen, Yueyue Zhang, Weijie Yuan, Bin Han

**Affiliations:** ^1^Department of Psychiatry, Huzhou Third People’s Hospital, The Affiliated Hospital of Huzhou University, Huzhou, China; ^2^Department of Nephrology, Shanghai General Hospital, Shanghai Jiaotong University School of Medicine, Shanghai, China

**Keywords:** apathy, dialysis, mortality, hospitalization, cox regression analyses

## Abstract

**Background:**

Patients receiving maintenance dialysis experience increased rates of hospitalization and mortality. Apathy is associated with reduced quality of life and increased hospitalization, institutionalization, and death. Whether apathy contributes to poor outcomes in population undergoing maintenance dialysis remain unknown.

**Methods:**

We conducted a prospective cohort study of maintenance dialysis population who were consecutively recruited at the Dialysis Center of Shanghai General Hospital between July 2017 and August 2018 and were followed up for 3 year. Apathy status was measured by the Apathy Evaluation Scale. The study outcomes were the occurrence of death and first hospitalization.

**Results:**

A total of 647 participants included in this study, 274 (42.3%) had a current apathy and 373 (57.7%) were not. During the follow-up period, 394 (60.9%) were hospitalized, and 169 (26.1%) died. Kaplan–Meier analysis showed that the risks of hospitalization and mortality were significantly higher in individuals with apathy than in those without apathy (both *p* < 0.001). Apathy at baseline was associated with hospitalization and death both in univariate analysis and in all multivariable models (all *p* < 0.001).

**Conclusion:**

Apathy was highly prevalent and independently correlated with an increased risk of poor outcomes in patients with maintenance dialysis.

## Introduction

Worldwide, more than 3 million patients with end-stage renal disease need maintenance dialysis to prolong their lifespan (1, 2) and the number is expected to double by 2030 (3). Increased hospitalizations and dialysis care for this population result in substantial medical expenditure, such that costs consumed $36.6bn in US in 2018 (4) and 40 billion renminbi in China in 2015 (5). Importantly, this dialysis population has an estimated 20 times higher probability of dying compared with the general population (6). Patients with hemodialysis have a significantly increased risk of death, which has been basically unchanged over the last 20 years (7). Hence, identifying modifiable risk factors of poor outcomes may help in administration of early interventions to reduce risk of hospitalization and mortality in patients with maintenance dialysis.

Apathy, defined as diminished initiative, lack of interest, limited emotional expression, and consequent reduced goal-directed behavior (8), is highly prevalent among community-dwelling older people, elderly hospitalized patients, and nursing home patients (9–11). Apathy is a distinct syndrome from depression (12) and has unique neuroanatomic correlation in the dorsolateral prefrontal cortex and subcortical structures (13). Despite some symptomatic overlap, appropriate assessment can clinically distinguish apathy from depression (14). Importantly, presence of apathy has been associated with significant disease progression, reduced quality of life, more hospitalization and institutionalization, and increased medical costs and mortality in community-dwelling older people, elderly hospitalized patients, and nursing home patients.

A recent study with small sample size shown that apathy was frequently observed among patients on chronic hemodialysis, with its prevalence of approximately 50% (15). Large studies are needed to confirm the prevalence of apathy in patients with maintenance dialysis. Currently, studies investigating the influence of apathy on clinical outcomes in individuals undergoing maintenance dialysis are lacking. A cohort of patients with maintenance dialysis was therefore prospectively studied to determine whether there was an association between presence of apathy, based on the Apathy Evaluation Scale, and hospitalization, or mortality during 3 year of follow-up.

## Methods

### Study design and population

We conducted a prospective cohort study of patients treated with maintenance dialysis in the Dialysis Center of Shanghai General Hospital Affiliated to Shanghai Jiaotong University School of Medicine in Shanghai, China. Patients were consecutively recruited in the period of 1 July 2017 and 31 August 2018 and were followed up from the baseline to the date of death, or 31 August 2021. Inclusion criteria was having a dialysis vintage of ≥90 days. Exclusion criteria were as follows: (a) age < 18 years; (b) a life-threatening comorbidity such as cancer; (c) individuals who were unable to complete the self-reported questionnaires; and (d) patients missing clinical information. The study protocol adhering to the Declaration of Helsinki was approved by the Ethics Committees of Shanghai General Hospital. Written informed consents were acquired from all participants.

### Demographic and clinical data

The following demographic and clinical data at baseline were collected from the electronic medical records of the hospital: age, sex, body weight and height, marital status, primary kidney disease, dialysis modality and vintage, comorbidities, and laboratory parameters. Body mass index was calculated as weight in kilograms divided by height in meters squared (kg/m^2^). Primary kidney disease was ascertained according to the European Renal Association-European Dialysis and Transplant Association (ERA-EDTA) coding system. The level of comorbidities was classified according to the Charlson comorbidity index. Laboratory parameters included the serum concentration of albumin, hemoglobin, calcium, phosphorus, intact parathyroid hormone (PTH), and Kt/V.

### Measures

We assessed apathy using the self-rated version of the Apathy Evaluation Scale (AES-S) (16), a well-validated instrument with the ability to discriminate from depression and anxiety (17). The 18-item scale measures apathy in cognitive, emotional, and behavioral domains within the last 4 weeks on a 4-point Likert scale. The summed score ranges from 18 to 72, with higher scores reflecting more apathy. AES has satisfactory test–retest reliability (*r* = 0.76) and internal consistency (α = 0.86) (16). Apathy is defined as a score of 37 or more (16). Depression was assessed by Zung Self-rating Depression Scale (SDS) (18). It contains 20 items involving affective, psychological, and somatic features of depression on a 4-point Likert scale, with a summed score ranging from 20 to 80. Score of ≥50 indicates presence of depression (19). We measured cognitive function using the Montreal Cognitive Assessment (MoCA) with moderate specificity and high sensitivity in individuals on maintenance dialysis (20, 21). The total score of MoCA is ranged from 0 to 30 and the cut-off value for cognitive impairment is 26 (21). All measurements were administered by trained research staff blinded to the medical history of the patient.

### Outcomes

Participants were followed up for 3 year from apathy ascertainment. The study outcomes were the occurrence of death and first hospitalization. Event ascertainment was conducted by 2 adjudicators blinded to baseline apathy status. Information on death and hospitalizations were obtained from the electronic medical records every 6 months and were confirmed with the patients or their relatives. Individuals were censored at the time of transferring to other dialysis units or receiving kidney transplantation.

### Statistical analysis

Data are presented as median (interquartile range, IQR), mean ± standard deviation (SD), or percent frequency. Normal distribution was determined using Kolmogorov–Smirnov test. Comparison between groups was carried out using Pearson’s *χ*^2^ test for categorical variables and Student’s *t* test or Mann–Whitney *U* test for continuous variables. To test the associations of apathy with hospitalization and mortality in participants undergoing maintenance dialysis, we performed Kaplan–Meier analyses and Cox regression analyses. We first conducted univariate analyses and then adjusted for demographics, cause of kidney failure, Charlson comorbidity index, dialysis characteristics, and laboratory parameters (Model 1). In order to examine independence from depression or cognitive impairment, we separately adjusted for depression (Model 2) or separately adjusted for cognitive impairment (Model 3), respectively. Results were reported with hazard ratios (HR) and 95% confidence intervals (CI). Cox proportional hazard model assumption was checked by using Scaled Schoenfeld residual test. Statistical analyses were conducted using SPSS 22.0 (Chicago, IL, United States). A two-tailed *p* < 0.05 were considered statistically significant.

## Results

### Baseline characteristics

**Figure 1:**
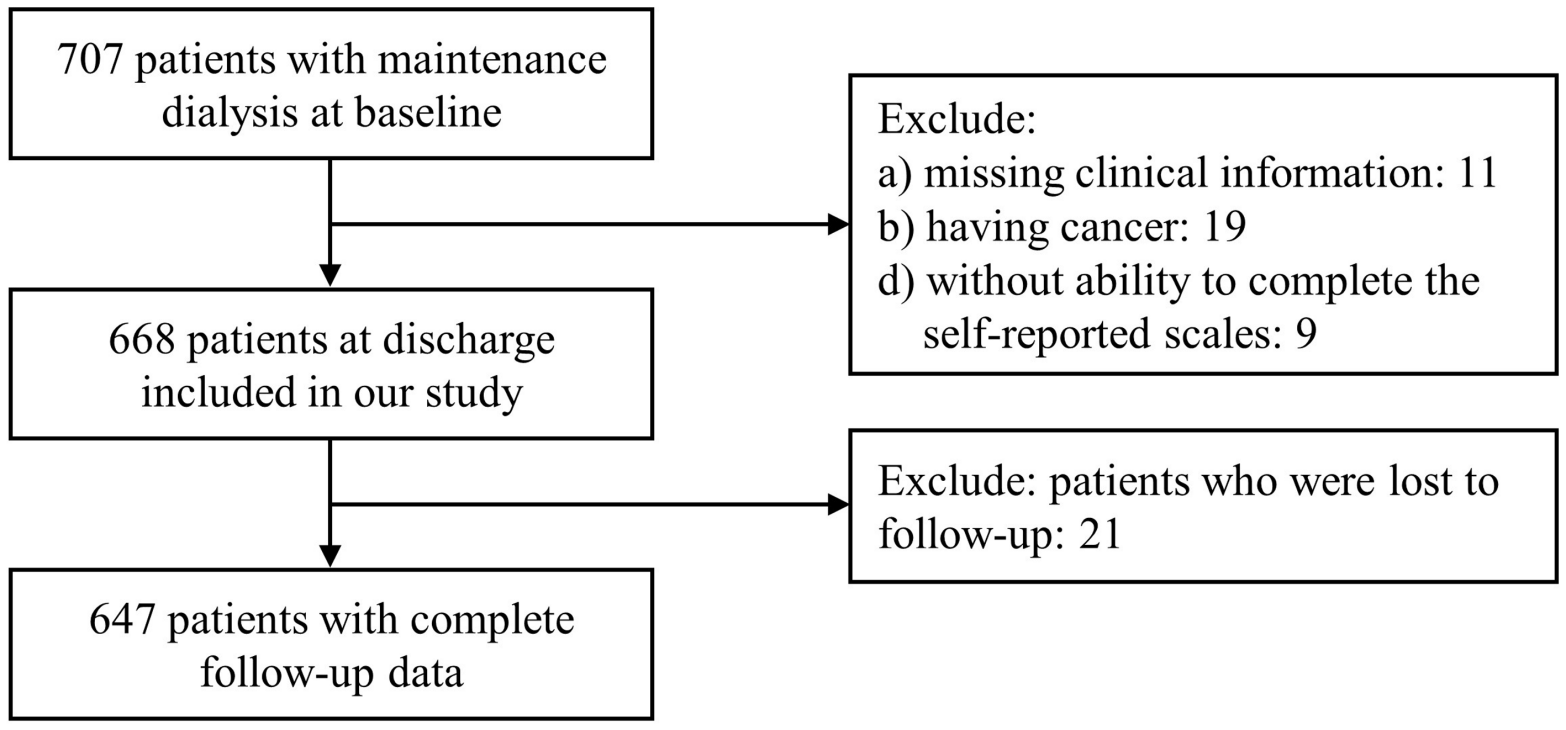
Inclusion diagram.

A total of 707 patients on maintenance dialysis, 60 were excluded this cohort study: 11 with missing clinical information, 19 having cancer, 9 who were unable to complete the self-reported questionnaires, and 21 who were lost to follow-up (Figure 1). There were no significant differences in regard to demographic and clinical data between the final cohort (*n* = 647) and those excluded. Of 647 patients who had a median dialysis vintage of 7 (IQR, 4–10) years, were eligible for inclusion in this cohort study. At baseline, participants had a mean (SD) age of 62.5 (6.6) years, 37.2% were female, 85.3% received HD, and 64.5% had a Charlson comorbidity index of 3 or more. The cohort had a median follow-up period of 3.2 (IQR, 2.1–3.5) years. Among participants, 274 (42.3%) had apathy and 373 (57.7%) were not. The apathy group at baseline were older, had longer dialysis vintage, and were less likely to be married compared to the group without apathy. According to the cutoff values, 54.7% had depression and 46.1% had cognitive impairment. Patients with apathy were significantly more likely to have a higher prevalence of depression or cognitive impairment as those without apathy. There were no significant between-group differences in regard to sex, body mass index, primary kidney disease, Charlson comorbidity index, or laboratory values between the two groups (Table 1).

**Table 1:** Baseline characteristics of dialysis patients by apathy status.

Characteristics	Apathy (*n* = 274)	No apathy (*n* = 373)	*p*-value
Demographics			
Age (years)	63.34 ± 7.33	61.83 ± 5.92	0.005
Female	108 (39.4)	133 (35.7)	0.328
Body mass index (kg/m^2^)	22.7 (16.4–27.7)	22.7 (15.1–26.5)	0.311
Married/life partner	263 (70.5)	217 (79.2)	0.013
Primary kidney disease			
Diabetes	87 (31.8)	114 (30.6)	0.747
Hypertension	79 (28.8)	94 (25.2)	0.302
Glomerulonephritis	62 (19.4)	85 (22.8)	0.273
Polycystic kidney	16 (5.8)	20 (5.4)	0.793
Other	36 (13.1)	42 (11.3)	0.468
Charlson comorbidity index			0.737
1–2	94 (34.3)	136 (36.5)	
3–4	105 (38.3)	132 (35.4)	
≥5	75 (27.4)	105 (28.2)	
Dialysis characteristics			
Hemodialysis	233 (85.0)	319 (85.5)	0.863
Vintage (years)	8 (5–11)	6 (4–10)	0.016
Single-pool Kt/V	1.56 ± 0.6	1.51 ± 0.44	0.242
Laboratory values			
Albumin (g/L)	34.42 ± 7.4	35.05 ± 8.91	0.34
Hemoglobin (g/L)	111.8 ± 26.1	110.8 ± 25.34	0.607
Calcium (mmol/L)	2.46 ± 0.23	2.46 ± 0.22	0.725
Phosphate (mmol/L)	1.66 (1.00–2.66)	1.66 (1.06–2.46)	0.436
Intact PTH (pg/ml)	250.8 (205.2–285.0)	250.8 (210.9–279.3)	0.937
Neuropsychiatric tests			
Cognitive impairment (MoCA < 26)	158 (59.8)	140 (37.5)	<0.001
Depression (SDS ≥ 50)	194 (70.8)	160 (42.9)	<0.001

Quantitative values are presented as medians (interquartile ranges) or mean ± standard deviation (SD), and qualitative values are presented as number of participants/percentage. PTH, parathyroid hormone; MoCA, Montreal Cognitive Assessment; SDS, Zung Self-rating Depression Scale.

### Association between apathy and hospitalization

**Figure 2:**
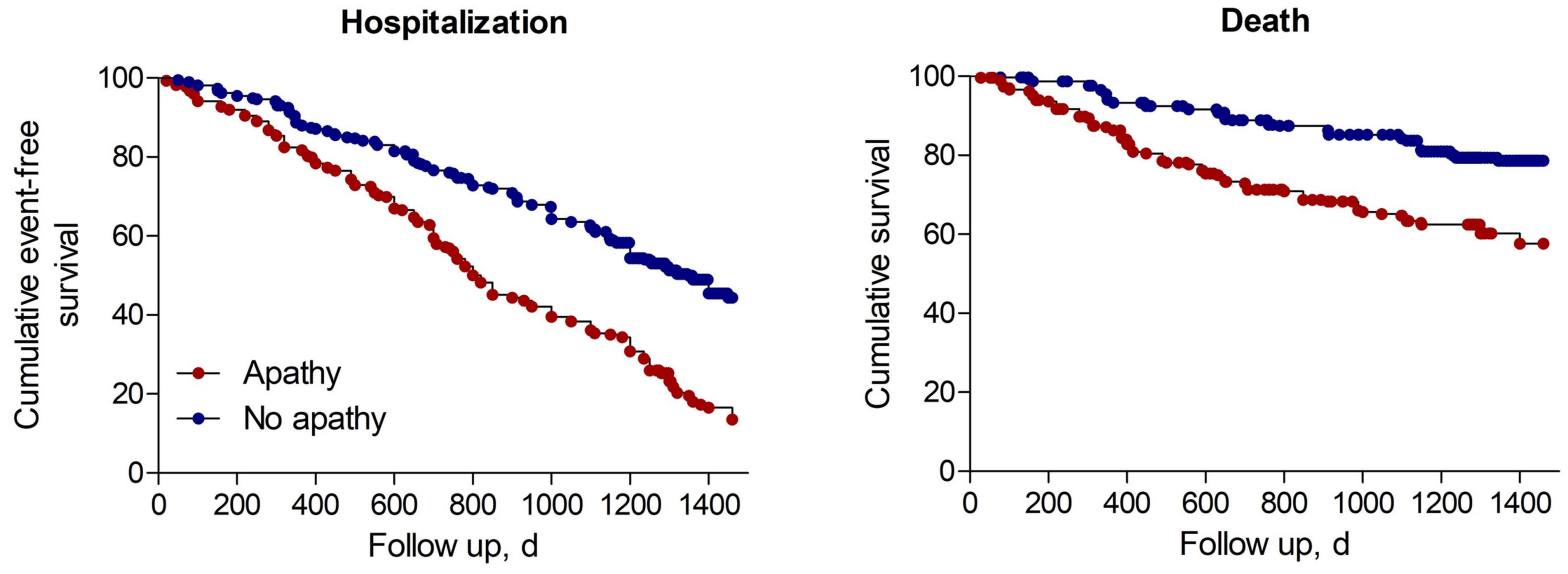
Cumulative event-free survival (Kaplan-Meyer curves) for hospitalization or death in dialysis patients.

During the follow-up period, 394 (60.9%) were hospitalized, ranging from 50.1% in the group without apathy to 55.5% in the group with apathy (*p* < 0.001). Kaplan–Meier analysis demonstrated that the risk of hospitalization was significantly higher in individuals with apathy than in those without apathy (*p* < 0.001 by log rank test, Figure 2). In univariate Cox regression analyses, apathy was significantly associated with an increased risk of hospitalization (HR 2.141, 95% CI 1.753–2.615, *p* < 0.001). After adjusting for demographics, primary kidney disease, Charlson comorbidity index, dialysis characteristics, and laboratory values, participants with apathy had twice the risk of being hospitalized compared with those without apathy (HR 2.101, 95% CI 1.707–2.586, *p* < 0.001). The association remained significant in the model adjusted additionally for depression (HR 1.815, 95% CI 1.147–2.227, *p* < 0.001) or in the model adjusted additionally for cognitive impairment (HR 2.095, 95% CI 1.713–2.562, *p* < 0.001; Table 2).

**Table 2:** Risk of hospitalization associated with apathy in unadjusted and adjusted Cox model.

	HR	95% CI	*p*-value
Univariate analysis	2.141	1.753–2.615	<0.001
Multivariate analysis			
Model 1^a^	2.101	1.707–2.586	<0.001
Model 2^b^	1.815	1.479–2.227	<0.001
Model 3^c^	2.095	1.713–2.562	<0.001

HR, hazard ratios; CI, confidence intervals.

a Model 1 is adjusted for demographics, cause of kidney failure, Charlson comorbidity index, dialysis characteristics, and laboratory values.

b Model 2 is adjusted additionally for depression.

c Model 3 is adjusted additionally for cognitive impairment.

### Association between apathy and mortality

During the follow-up period, 169 (26.1%) were died, ranging from 18.2% in the group without apathy to 36.5% in the group with apathy (*p* < 0.001). Kaplan–Meier analysis showed that the risk of death was significantly higher in individuals with apathy than in those without apathy (*p* < 0.001 by log rank test, Figure 2). In univariate Cox regression analyses, apathy was significantly associated with an increased risk of mortality (HR 2.508, 95% CI 1.840–3.419, *p* < 0.001). After adjusting for demographics, primary kidney disease, Charlson comorbidity index, dialysis characteristics, and laboratory values, participants with apathy were twice as likely to be died compared with those without apathy (HR 2.158, 95% CI 1.560–2.985, *p* < 0.001). The association remained robust in the model adjusted additionally for depression (HR 2.118, 95% CI 1.541–2.913, *p* < 0.001) or in the model adjusted additionally for cognitive impairment (HR 2.283, 95% CI 1.670–3.121, *p* < 0.001; Table 3).

**Table 3:** Risk of death associated with apathy in unadjusted and adjusted Cox model.

	HR	95% CI	*p*-value
Univariate analysis	2.508	1.840–3.419	<0.001
Multivariate analysis			
Model 1^a^	2.158	1.560–2.985	<0.001
Model 2^b^	2.118	1.541–2.913	<0.001
Model 3^c^	2.283	1.670–3.121	<0.001

HR, hazard ratios; CI, confidence intervals.

a Model 1 is adjusted for demographics, cause of kidney failure, Charlson comorbidity index, dialysis characteristics, and laboratory values.

b Model 2 is adjusted additionally for depression.

c Model 3 is adjusted additionally for cognitive impairment.

## Discussion

The new finding in this prospective cohort study of participants undergoing maintenance dialysis is that presence of an apathy ascertained by AES-S predicts hospitalization, or death within 3 year of apathy ascertainment. To our knowledge, this is the first report of such an association in this patient population. Patients with apathy were twice as likely to be hospitalized and more than 2 times as likely to die as those without apathy. The increased risks were robust and independent of demographics, cause of kidney failure, Charlson comorbidity index, dialysis characteristics, laboratory values, depression and cognitive impairment.

Growing evidence support the note that apathy is tightly linked with poor clinical outcomes. In a population-based and multi-center study of cognitively impaired older people, apathy was associated with 3.1 times higher death than those without apathy (22). The presence of apathy with Alzheimer’s disease is linked with functional impairment, decreased activities of daily living, more morbidity, and increased mortality (23). Poststroke apathy was associated with poor functional rehabilitation and increased mortality risk (24, 25). Apathetic patients with Parkinson’s disease showed excess disability, reduced quality of life, increased hospitalization and institutionalization (26, 27). The association of apathy with adverse prognosis was also observed among community-dwelling older people, elderly hospitalized patients, and nursing home patients (9–11).

The exact mechanisms underlying the association between presence of apathy and poor outcomes have not been elucidated. Apathetic patients with Parkinson’s disease exhibited serotonergic degeneration in the brainstem (28). In infancy, serotonergic defects in the brainstem has been linked with an increased risk of sudden death (29). Similarly, a link between serotonin and death been found in epilepsy patients and in animal models (30). It may therefore be mediating mechanism via which apathy in dialysis individuals can affect prognosis. Additionally, emerging study of patients with early Parkinson’s disease demonstrated that the presence of apathy correlated to peripheral inflammation (31) which amplifies the risks of hospitalization and mortality in hemodialysis patients (32). The behavioral mechanisms to explain the association of apathy with poor outcomes include nonadherence to medical treatment and healthy lifestyle observed in patients with apathy (33, 34). Diminished behavioral adherence is consequently related with increased mortality in patients with hemodialysis (35). Hence, the influence of apathy on poor outcomes is likely the result of bio-psycho-social interaction.

Although there are currently no approved pharmacologic treatments for apathy, several nonpharmacological approaches to treating it have been recommended, including exercise, music therapy, multisensory stimulation, brain stimulation, pet therapy, and the use of digital therapies (36). There are several limitations in this study. First, all individuals were of Chinese descent only, which limited generalization of the current results. Second, patients who refused to give written informed consent excluded from this cohort study. We tried to diminished the potential bias by enrolling participants consecutively. Third, we ascertained presence of apathy using the AES-S, which may not be as sensitive as an in-depth psychiatric interview based on the diagnostic criteria for apathy in neurocognitive disorders (8). But the AES-S (16) used in this study is a well-validated tool with the ability to discriminate from depression and anxiety (17). Finally, participants in our study were enrolled from a single dialysis unit, thereby limiting the generalizability to the other population with maintenance dialysis.

## Conclusion

Despite the limitations mentioned above, our study indicates that apathy was highly prevalent and independently correlated to hospitalization, or death in patients with maintenance dialysis. Apathy should be one of the primary concerns for clinicians who provide treatment to chronic dialysis patients. There is a major need in future clinical trials to explore the treatment of apathy in this patient population, and to determine whether a positive effect on apathy will improve clinical outcomes and quality of life.

## Data availability statement

The raw data supporting the conclusions of this article will be made available by the authors, without undue reservation.

## Ethics statement

These studies involving human participants were reviewed and approved by the Ethics Committees of Shanghai General Hospital. Written informed consents were acquired from all participants.

## Author contributions

BH and WY conceptualized the study, provided supervision, and reviewed and edited the manuscript. YF, LC, BH, and YZ were responsible for data acquisition and data analysis. YF, LC, YZ, WY, and BH were responsible for data interpretation. YF and LC wrote the original draft. All authors contributed to the article and approved the submitted version.

## Funding

This work was supported by the National Natural Science Foundation of China (81970624).

## Conflict of interest

The authors declare that the research was conducted in the absence of any commercial or financial relationships that could be construed as a potential conflict of interest.

## Publisher’s note

All claims expressed in this article are solely those of the authors and do not necessarily represent those of their affiliated organizations, or those of the publisher, the editors and the reviewers. Any product that may be evaluated in this article, or claim that may be made by its manufacturer, is not guaranteed or endorsed by the publisher.
